# A Comparison of the Oral Microbiota in Healthy Dogs and Dogs with Oral Tumors

**DOI:** 10.3390/ani13233594

**Published:** 2023-11-21

**Authors:** Anja Lisjak, Bruna Correa Lopes, Rachel Pilla, Ana Nemec, Jan S. Suchodolski, Nataša Tozon

**Affiliations:** 1 Small Animal Clinic, Veterinary Faculty, University of Ljubljana, 1000 Ljubljana, Slovenia; ana.nemec@vf.uni-lj.si (A.N.); natasa.tozon@vf.uni-lj.si (N.T.); 2 Gastrointestinal Laboratory, Department of Small Animal Clinical Sciences, College of Veterinary Medicine & Biomedical Sciences, Texas A&M University, College Station, TX 77843, USA; brunalopes@tamu.edu (B.C.L.); rpilla@cvm.tamu.edu (R.P.); jsuchodolski@cvm.tamu.edu (J.S.S.)

**Keywords:** oral microbiota, DNA shotgun sequencing, oral tumors, oral swab, biopsy, dogs

## Abstract

**Simple Summary:**

The oral microbiota in dogs constitutes a complex community. In recent years, oral microbiota has caught the interest of many as it is thought to play a key role in systemic diseases, including cancer. In this study, our objective was to delineate the core oral microbiome of healthy dogs and to identify any differences between healthy dogs and those with oral tumors. Oral swabs, representing all oral niches, were taken from both groups and analyzed using DNA shotgun sequencing. The core oral microbiota of healthy dogs was determined, consisting of a total of 67 bacterial species. Significantly higher species richness and evenness were observed in healthy dogs, while there was no significant difference in community composition between the healthy dogs and the dogs with oral tumors. To our knowledge, our study is the first to use DNA metagenomic sequencing to determine a core oral microbiome. Our study provides a novel approach and insight into the composition of the oral microbiota of dogs.

**Abstract:**

The aim of this study was to further describe the oral microbiota of healthy dogs by DNA shotgun sequencing and compare those to dogs with oral tumors. Oral swabs (representative of all niches of the oral cavity) were collected from healthy dogs (n = 24) and from dogs with different oral tumors (n = 7). DNA was extracted from the swabs and shotgun metagenomic sequencing was performed. Only minor differences in microbiota composition were observed between the two groups. At the phylum level, the Bacteroidota, Proteobacteria, Actinobacteriota, Desulfobacterota and Firmicutes were most abundant in both groups. Observed Operational Taxonomic Units—OTUs (species richness) was significantly higher in the healthy patients, but there was no significant difference in the Shannon diversity index between the groups. No significant difference was found in beta diversity between the groups. The core oral microbiota consisted of 67 bacterial species that were identified in all 24 healthy dogs. Our study provides further insight into the composition of the oral microbiota of healthy dogs and in dogs with oral tumors.

## 1. Introduction

The microbiota of the oral cavity is highly diverse. In humans, the mouth of an average adult harbors 50–100 million bacteria and has been estimated to comprise up to approximately 700 different species based on 16S rRNA sequencing [1]. Recent studies in dogs have shown that the oral microbiota of dogs likely comprises similar numbers, but the microbial populations differ from that found in humans [2]. A variety of human oral diseases, including periodontitis, dental caries, endodontic disease, and dry socket have been associated with changes in the oral microbiota. It has also been found that the oral microbiota can serve as a biomarker for cardiovascular diseases, bacterial pneumonia, and pancreatic cancer [1,3]. While primary endodontic infections in humans are well documented, data obtained in veterinary dentistry are mostly from culture-based studies [4,5,6]. In a study examining the microbiome of naturally occurring primary endodontic infections in dogs, using 16S rRNA sequencing, the authors confirmed that there was no significant difference between sulcal and endodontic samples. Both sulcal and endodontic samples were found to contain a mixed microbiota with comparable richness and diversity, and were primarily composed of the same phyla. The most abundant phyla in all the samples were Bacteroidota, Proteobacteria and Firmicutes [7].

The NIH (United States National Institutes of Health) Roadmap for Medical Research recently launched the Human Microbiome Project with the goal of comprehensively characterizing the human microbiota and analyzing its role in health and disease. Initial efforts included the sampling of multiple sites in healthy volunteers to determine whether people share basic profiles of microbial diversity [8]. Microbial diversity manifests differently in different ecological niches of the body. For example, greater diversity is expected in the gut, but this may be altered in dysbiotic conditions [9]. The Human Oral Microbiome Database (HOMD) provides a collection of genome sequences of oral bacteria and a detailed resource with descriptions of oral bacterial taxa, a 16S rRNA gene identification tool [10]. It is a unique database launched in 2010 by the National Institute of Dental and Craniofacial Research to provide information on culturable and non-culturable isolates from the oral cavity [11]. There are currently no such databases in veterinary medicine, although the canine oral microbiome was first defined over a decade ago [12,13,14,15] and was refined later [16,17,18,19].

Data from human studies also point towards the possible impact of oral microbiota on carcinogenesis with three possible mechanisms of action [20]. The first is bacterial stimulation of chronic inflammation. The inflammatory mediators produced in this process cause or facilitate cell proliferation, mutagenesis, oncogene activation and angiogenesis. Secondly, bacteria can influence carcinogenesis by directly affecting cell proliferation, cytoskeletal restructuring, nuclear factor kappa (NF-ƘB, a protein transcription factor) activation and inhibition of cellular apoptosis. And, thirdly, bacteria produce certain substances that can be carcinogenic [20]. It has been reported that *Fusobacterium nucleatum* that can be found in the oral and intestinal microbiota of humans can trigger the development of colon cancer. *Fusobacterium nucleatum* does not settle on healthy tissue, but on colorectal tumors, and there they begin to actively multiply, causing the disease to progress [21]. Many bacterial strains have been recognized and strongly associated with the development of oral cancer and the dysbiosis of the oral microbiota and oral mucosal homeostasis, which may also represent modifiable risk factors for the development of oral squamous cell carcinoma (OSCC) in humans. [22]. Several species such as *Capnocytophaga gingivalis*, *Fusobacterium* sp., *Streptococcus* sp., *Peptostreptococcus* sp., *Porphyromonas gingivalis*, and *Prevotella* sp. have been strongly associated with oral carcinomas, as these bacteria can promote inflammation, cell proliferation, and the production of oncogenic substances [23]. In humans, decreases in Firmicutes and increases in Fusobacteria were described in OSCC samples [24,25], and significantly higher concentrations of *Peptostreptococcus*, *Fusobacterium*, *Prevotella* (especially *Prevotella melaninogenica*), *Porphyromonas*, *Veillonella* (mainly *Veillonella parvula*), *Haemophilus*, *Rothia*, and *Streptococcus* were found [24,25,26,27,28,29]. In recent years, evidence of potentially carcinogenic properties of two oral bacteria in in vitro and animal models has emerged. Both *Porphyromonas gingivalis* and *Fusobacterium nucleatum* can induce inflammatory cytokine production, cell proliferation and cellular invasion in human OSCC via different mechanisms [30,31,32,33]. *Porphyromonas gingivalis* has been found to be responsible for inducing the production of interleukins, tumor necrosis factor-alpha (TNF-α), and matrix metalloproteinases (MMPs), as well as for inhibiting apoptosis [30]. It also prevents the activity of the tumor suppressor gene *p53* [30,32]. Prolonged exposure to *Porphyromonas gingivalis* has also been shown to increase the invasiveness of OSCC [30].

While there is evidence on the association between microbiota and neoplastic diseases in dogs [33,34,35,36,37], data on oral bacteria contribution to tumorigenesis in animals is limited. In a study that investigated the risk factors and etiology of the canine oral mucosal melanoma (OMM) in dogs, 16S rRNA sequencing was used for microbiome analyses and found that the bacteria *Tannerella forsythia* and *Porphyromonas gingivalis* were found in significantly higher amounts in dogs with OMM and their presence could be considered a risk factor for the development of OMM in dogs [37]. Stashenko et al. [35] colonized germ-free mice with different oral microbiomes and exposed them to a carcinogenic 4-nitroquinoline-1-oxide (4-NQO) agent. Mice with the oral microbiome and 4-NQO had more and larger OSCCs compared to the controls treated with 4-NQO alone [35]. In general, carcinogenic microbial infection and microbiota dysbiosis are associated with tumorigenesis, and intratumoral and local tumor microbiota also contribute significantly to tumor progression. However, the causal relationship between these two factors needs further investigation [38].

A recent study focused on identifying the bacteria living in the oral mucosa of dogs using culture-based methods and 16S rRNA sequencing, and also measured the C-reactive protein (CRP) and compared blood profiles. Swabs were taken from the tongue mucosa and the hard palate mucosa, and three groups of dogs were compared (dogs with and without oral tumors, and those presenting metastasis). The most common genera in the group without oral tumors were *Neisseria* spp., *Pasteurella* spp. and *Staphylococcus* spp., while *Escherichia* spp. and *Shigella* spp. were not isolated in these dogs. *Neisseria* spp. and *Pasteurella* spp. decreased in both oral tumor groups. The observed differences in oral bacteria, the increase in CRP, the CRP–albumin ratio (CAR), and the neutrophil–lymphocyte ratio (NLR) in both oral tumor groups suggested that bacteria and oral tumors may be related, and that bacteria may play a role in carcinogenesis [39]. Nonetheless, it is important to acknowledge that carcinogenesis is a multifaceted process influenced by factors beyond the microbiome. Further research is needed into the intricate the interplay among the immune system, environmental influence, and the microbiome and their effects on carcinogenesis.

To our knowledge, most previous studies on the oral microbiota of dogs [2,7,16,18,19] have been based on 16S rRNA gene amplicon sequencing, in which a small region of a ribosomal sequence is amplified and sequenced. Metagenomic shotgun sequencing (MGS) used in our study allows whole-genome sequencing and provides more information about the microbial community with higher taxonomic resolution than 16S rRNA gene sequencing. Therefore, our study can help build the database of the oral microbiota of healthy dogs and provide insight into its changes associated with oral cancer in dogs.

## 2. Materials and Methods

### 2.1. Study Population and Sample Collection

Oral swabs were collected from client-owned healthy dogs and dogs with oral tumors. All samples were collected at the Small Animal Clinic, Veterinary Faculty, University of Ljubljana, Ljubljana, Slovenia. The following inclusion criteria applied to all dogs: for at least 2 months prior to swab collection, dogs must not have been treated with antibiotics, proton pump inhibitors or corticosteroids. All owners had to complete a questionnaire about the dogs’ health status, activity, medication intake, and diet, and sign a written consent form (Supplementary Table S1). Before sampling, full physical exams, and blood analyses (complete blood count, urea, creatinine, alkaline phosphatase, and alanine transaminase) were performed. Oral swabs of the tumor patients were taken under general anesthesia just prior to taking a biopsy or performing the diagnostic workup or treatment for the oral tumor. The anesthetic protocol was standardized. Each dog received medetomidine/dexmedetomidine in combination with methadone as premedication. Fifteen minutes later, anesthesia was induced with propofol and maintained with either sevoflurane or isoflurane. In healthy dogs, swabs were obtained on awake patients. For both healthy control group and tumor patients, swabs were taken in the same manner to represent microbiota of different niches of the oral cavity. With the same swab, we sampled the buccal mucosa and teeth (starting with quadrant 1 and following with quadrants 2, 3 and 4), tongue and hard and soft palate (in this sequence). For the buccal mucosa and teeth, each quadrant was swabbed 5 times (five rostral–caudal strokes) before moving to the next quadrant. The tongue and the hard and soft palate were each swabbed 5 times (five rostral–caudal strokes). After sampling, the swab was immediately transferred to the lysis buffer (Mobio Power Soil DNA Extraction kit; MoBio Laboratories, Inc., Carlsbad, CA, USA) and stored at −80 °C.

### 2.2. Deepseq^TM^ Shotgun Metagenomic Sequencing

The MoBio PowerSoil^®^ DNA isolation kit (MoBio Laboratories, Inc., Carlsbad, CA, USA) was used to extract the microbial DNA from the oral samples. The DNA was quantified by the Quant-It^TM^ PicoGreen^TM^ dsDNA assay kit (Invitrogen^TM^, Waltham, MA, USA). In the preparation of the sequencing libraries, the Nextera^®^ XT DNA Library Preparation Kit (Illumina Inc., San Diego, CA, USA) was used before the libraries were pooled. After the library preparation, solid-phase reversible immobilization (SPRI) bead purification and concentration were processed using Sera-Mag^TM^ SpeedBeads Carboxylate-Modified Magnetic Particles (Cytiva^TM^ Life Sciences, Marlborough, MA, USA). The resulting pooled libraries were denatured by Sodium Hydroxide (NaOH) before being diluted and spiked by 2% PhiX control.

The metagenomic sequencing was conducted on an Illumina NovaSeq^TM^ 6000 System (Illumina Inc., San Diego, CA, USA) using a paired end 2 × 150 base pair (bp) read chemistry. Subsequently, the data was multiplexed on the sequencer, converted to FASTQ format files, and filtered for poor quality (Q-score < 30) and short length (<50). Adapter sequences were removed, and all reads were trimmed to a maximum length of 100 bp before the alignment.

The raw sequences were made using National Center of Biotechnology Information (NCBI) Sequence Read Archive and subsequently processed using established pipelines. For taxonomic classification, FASTA sequences were aligned, making a curated database which included all representative genomes in the NCBI RefSeq, a representative genome collection for prokaryotes with additional manually curated dog-specific Metagenomically Assembled Genomes (MAGs) and isolated genomes [40]. Only high-quality MAGs, characterized by completeness greater than 90% and contamination lower than 5% (as determined by CheckM) were considered.

Alignments were performed at 97% identity and compared to genomes of reference. Using fully gapped alignment with BURST, each input sequence was compared to every reference sequence in the Diversigen^®^ DivDB-Dog database. In case of ties, preference was given to minimize the overall number of unique Operational Taxonomic Units (OTUs). The input sequences were assigned the lowest common ancestor for taxonomy, a classification compatible with no less than 80% of the reference sequences. Taxonomies were based on Genome Taxonomy Database (GTDB r95). Samples containing fewer than 10,000 sequences were excluded from the analysis. Additionally, OTUs accounting for less than one million of all species-level markers, as well as OTUs with less than 0.01% of their unique genome regions matching and <0.1% of the whole genome, were not included in the study.

### 2.3. Statistical Analysis

Statistical analyses were performed using GraphPad Prism software (Version 9.4.1). The Shapiro–Wilk test was used for the normality assessment of the data. Unpaired *t* test and Mann–Whitney test were employed to assess the age, sex, and weight differences within the two groups: healthy dogs and those with oral tumors. Descriptive statistics on parametric data were reported as mean, minimum, and maximum values.

Regarding results obtained by the sequencing analysis, the Mann–Whitney test was used for within-group comparisons. The microbial relative abundances were screened for differences among healthy and oral tumor groups with *p*-values adjusted for multiple hypothesis testing using the Benjamini and Hochberg false discovery rate (FDR). Statistical significance was set at *p*-value < 0.5. For downstream analysis, normalized and filtered tables were used in QIIME2 [41]. Alpha diversity was evaluated through the Shannon–Wiener index and Chao1 index, and observed Operational Taxonomic Units. Beta diversity was evaluated with the analysis of similarities test (ANOSIM) by Primer 7 (Plymouth Routines in Multivariate Ecological Research Statistical Software, v7.0.13) using the Bray–Curtis dissimilarity.

## 3. Results

### 3.1. Study Population

Thirty-one client-owned dogs of different breeds participated in this study (Supplementary Table S2). The healthy control group consisted of 24 clinically healthy dogs, between 24 and 172 months of age, between 3 and 42 kg of weight, including ten intact males, one castrated male, five intact females and eight spayed females; the breeds included one German Pointer, one Giant Schnauzer, one Airedale terrier, one Cavalier King Charles Spaniel, one English Cocker Spaniel, two Tibetan Terriers, one Whippet, three German Spitzes, one Pomeranian, two Lagotto Romagnolos, one Beagle, one German Shepherd, one Russian Wolfhound, one Labrador Retriever, three French Bulldogs, one Keeshond and two German boxers. The group of oral tumor patients consisted of seven dogs between 46 and 143 months old, between 6 and 50 kg of weight, and included one intact male, two intact females and four spayed females; the breeds included one American bulldog, one Shih-tzu, one American Staffordshire Terrier, two crossbreeds, one Labrador Retriever and one Chihuahua. No statistical differences were observed between groups according to age, weight, and sex (Table 1).

All dogs from both groups had hematological results, kidney parameters and liver enzymes in the normal range and showed no signs of gastrointestinal disease. The oral tumor group included seven dogs with different histologically confirmed types of oral tumors: oral melanoma (n = 2), amelanotic melanoma (n = 1), fibrosarcoma (n = 2), osteosarcoma (n = 1), and plasmacytoma (n = 1). Dogs in this study were fed mainly commercial food: dry (11/24 healthy and 1/7 oral tumor) and a combination of dry and wet food (10/24 healthy and 5/7 oral tumor). Few dogs were fed home-cooked food (2/24 healthy and 1/7 oral tumor). None of the included dogs ate wet food only.

### 3.2. Oral Microbiota of Healthy Dogs

A total of 21,385,716 quality sequences were detected within a total of 1687 observations (Min 49,505; Max 2,691,669; Median 601,754; Mean 689,861.806; Standard Deviation 571,358.559). The samples were rarefied to a sequencing depth of 49,504 sequences per sample. The distribution was across 12 phyla. The predominant bacterial phyla in oral samples from healthy dogs were Bacteroidota, followed by Proteobacteria, Actinobacteria, Desulfobacterota and Firmicutes. Spirochaetota, Firmicutes_A, Fusobacteria, unclassified phylum from bacteria, Campylobacterota, Patescibacteria and Firmicutes_C were also present at lower frequencies (Table 2).

At the genus level, the ten most abundant were *Porphyromonas* A, followed by *Porphyromonas*, *Conchiformibius*, *Capnocytophaga*, *Neisseria*, *Pasteurella*, *Frederiksenia*, *Bergeyella*, *Histophilus* and *Desulfomicrobium* (Supplementary Table S3).

At the species level, *Porphyromonas A cangingivalis* was the most abundant bacterium, followed by *Porphyromonas gulae*, *Conchiformibius steedae*, *Porphyromonas A canoris*, and *Porphyromonas gingivicanis*. *Neisseria weaveri* and *Frederiksenia canicola* followed in lower abundances. Among the ten most abundant species, we also found *Capnocytophaga cynodegmi*, *Capnocytophaga canimorsus*, and *Capnocytophaga canis*. This was followed by species such as *Bergeyella zoohelcum*, *Pasteurella dagmatis*, *Histophilus haemoglobinophilus*, *Neisseria zoodegmatis*, *Desulfomicrobium orale*, and *Porphyromonas gingivalis* (Table 3).

### 3.3. Core Microbiota of Healthy Dogs

In our study, we defined the core microbiota as the bacterial species found in all healthy dogs. The core microbiota consisted of 67 bacterial species, with *Porphyromonas A cangingivalis*, *Porphyromonas gulae*, *Conchiformibius steedae*, *Porphyromonas A canoris*, *Porphyromonas gingivicanis*, *Neisseria weaveri*, *Frederiksenia canicola*, *Capnocytophaga cynodegmi*, *Capnocytophaga canimorsus*, *Capnocytophaga canis* and *Bergeyella zoohelcum* being the most abundant (Table 4).

### 3.4. Oral Microbiota of Dogs with Oral Tumors

**Figure 1:**
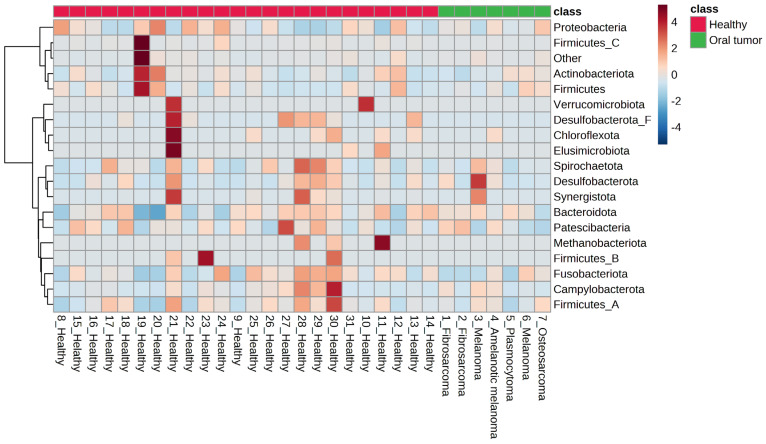
Heat map representing the relative abundance of the identified phyla in healthy dogs and those with oral tumors.

In the samples from dogs with oral tumors, the distribution of bacterial phyla was very similar to what was observed in healthy dogs and included the predominant bacterial phyla Bacteroidota, followed by Proteobacteria, Actinobacteria, Firmicutes, and Desulfobacterota. Spirochaetota, Firmicutes_A, Unclassified phylum from Bacteria, Campylobacterota, Fusobacteria, Patescibacteria, and Firmicutes_C were also present in lower abundance (Figure 1).

At the genus level, the ten most abundant were *Porphyromonas A*, followed by *Porphyromonas*, *Conchiformibius*, *Bergeyella*, *Capnocytophaga*, *Pasteurella*, *Frederiksenia*, and *Neisseria*, *Histophilus*, and *Corynebacterium* (Supplementary Table S3).

At the species level of dogs with oral tumors, *Porphyromonas A cangingivalis* was the most common bacterium, followed by *Porphyromonas gulae*, *Conchiformibius steedae*, *Porphyromonas A canoris*, and *Bergeyella zoohelcum*. These were followed by the bacteria *Porphyromonas gingivicanis*, *Frederiksenia canicola*, *Capnocytophaga canimorsus*, *Pasteurella canis*, *Capnocytophaga cynodegmi*, and *Capnocytophaga canis*. In lower abundances, we also found *Histophilus haemoglobinophilus*, *Porphyromonas crevioricanis*, *Pasteurella multocida A*, and *undetermined Porphyromonas* (Supplementary Table S3).

### 3.5. Differences in the Oral Microbiota between Healthy Dogs and Dogs with Oral Tumors

**Figure 2:**
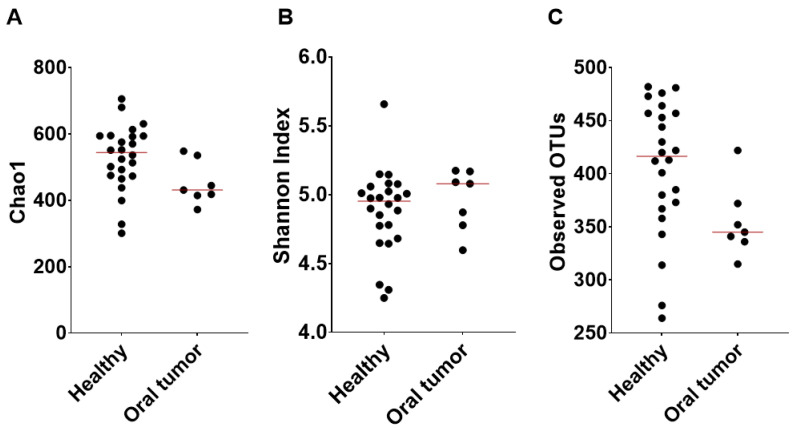
Alpha diversity metrics of healthy dogs and those with oral tumors: Chao1 (**A**), Shannon diversity index (**B**), and observed OTUs (**C**).

**Figure 3:**
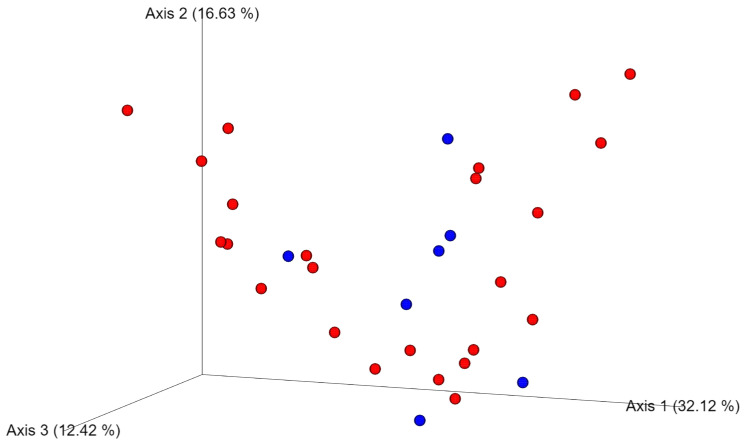
Principal Coordinates Analysis (PCoA) plot based on Bray–Curtis distances. Samples are color-coded based on the group classification. Red: healthy dogs. Blue: dogs with oral tumor. The ANOSIM analysis did not reveal clustering between groups (R = 0.126; P = 0.867).

The alpha diversity described by Chao 1 (P = 0.0292, Q = 0.0438) and the observed OTUs (species richness) (P = 0.0229, Q = 0.0438) were significantly higher in the healthy patients, whereas there was no significant difference in the Shannon diversity index (P = 0.3174, Q = 0.3174) compared with the oral tumor group (Figure 2). Principal Coordinates Analysis (PCoA) based on the Bray–Curtis distances (beta diversity) was not significantly different, as shown by the ANOSIM (R = 0.126, P = 0.867) (Figure 3). Differences between individual bacterial groups were analyzed using a Kruskal–Wallis test, and several bacterial taxa were significantly different between samples from healthy patients and oral tumor patients.

**Figure 4:**
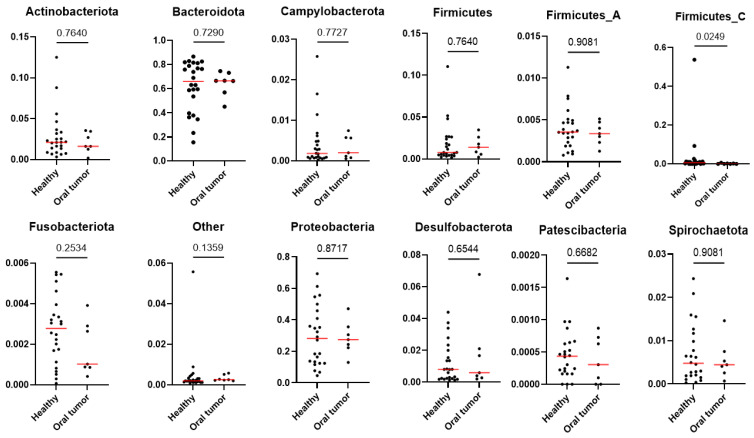
Differences in relative abundance at the phylum level. Difference noted only in the higher abundance of Firmicutes_C (P = 0.0249, Q = 0.2988) in the healthy group; however, statistical significance was lost after adjustment for multiple comparisons.

**Figure 5:**
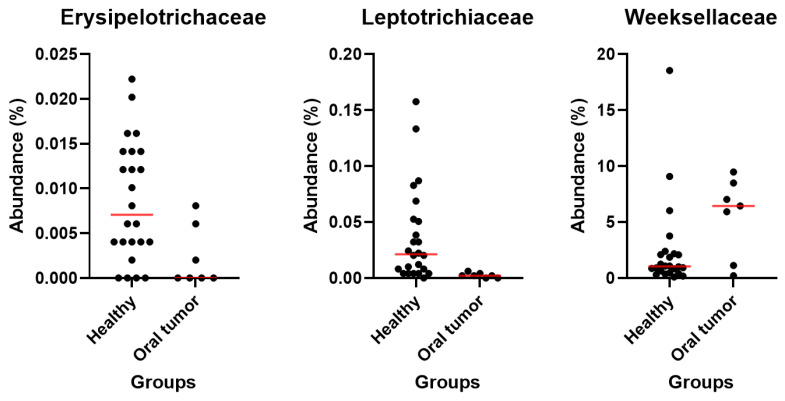
Differences in relative abundance at the family level: healthy dogs had higher abundance of *Erysipelotrichaceae* (P = 0.0201, Q = 0.4355) and *Leptotrichiaceae* (P = 0.0002, Q = 0.0130); *Weeksellaceae* family was found more abundant in dogs with oral tumors (P = 0.0473, Q = 0.5640).

At the phylum (Figure 4), class (Supplementary Figure S1) and order levels (Supplementary Figures S2 and S3), multiple comparison analysis demonstrates no statistically significant difference between the healthy and oral tumor groups. At the family level, we found differences in the abundance of *Erysipelotrichaceae* (P = 0.0201, Q = 0.4355) and *Leptotrichiaceae* (P = 0.0002, Q = 0.0130), which were more abundant in healthy dogs (Supplementary Figure S4). Lastly, the *Weeksellaceae* family showed a trend towards higher abundance in animals with oral tumors; however, it was no longer significant after adjustment for multiple comparisons (P = 0.0473, Q = 0.5640) (Figure 5).

Some differences in bacterial relative abundance were noted at the genus and species level; however, statistical significance was lost after adjustment for multiple comparisons. *Pauljensenia vaccimaxillae* (P = 0.0413, Q = 0.9239), *Prevotella* sp000467895 (P = 0.0110, Q = 0.5372), *Riemerella anatipestifer* (P = 0.0033, Q = 0.3907), *Alysiella* sp. (P = 0.0254, Q = 0.6766), *Kingella A denitrificans* (P = 0.0220, Q = 0.6766), *Biberstenia trehalosi* (P = 0.0040, Q = 0.3907), *Mannheimia* sp000521605 (P = 0.0083, Q = 0.4864), *Pasteurella canis* (P = 0.0194, Q = 0.6766), *Bergeyella zoohelcum* (P = 0.0473, Q = 0.9239) and *Haemophilus* sp. (P = 0.0250, Q = 0.6766) were more abundant in the oral tumor group, while *Corynebacterium freiburgense* (P = 0.0469, Q = 0.9239), *Fusobacterium* A sp. (P = 0.0219, Q = 0.6766), and *Streptobacillus felis* (P = 0.0026, Q = 0.3907) were more abundant in the healthy group.

## 4. Discussion

Our study used shotgun metagenomic sequencing to characterize the oral microbiome of dogs. This method allows more accurate identification of bacterial species compared to 16S rRNA gene sequencing, regardless of the type of sample analyzed [42]. To our knowledge, this is the first study evaluating and comparing the oral microbiota of healthy dogs and dogs with oral tumors applying strict inclusion criteria regarding medications that can significantly interfere with microbiota [43,44,45,46,47,48,49].

In a recent study, bacterial community profiles from four oral niches (soft tissue and hard tissue surfaces, as well as saliva) in healthy dogs were compared using a 16S rRNA gene sequencing approach. The four niches, except saliva, demonstrated similar microbial communities. *Bergeyella*, *Capnocytophaga*, *Neisseria*, *Porphyromonas* and *Fusobacterium* were observed amongst the core microbiota on the genus level [16]. In our study, all niches were represented in the swabs, and we observed the highest abundance of *Porphyromonas A*, *Porphyromonas*, *Conchiformibius*, *Capnocytophaga*, *Neisseria*, *Pasteurella*, *Frederiksenia* and *Bergeyella* at the genus level in healthy dogs, while *Bergeyella*, *Pasteurella* and *Frederiksenia* were present in higher abundances in dogs with oral tumors. At the species level, very similar bacterial taxa were found, but in different frequencies. In the study by Ruparell et al. [16], the most represented bacterial species were unclassified *Pasteurellaceae*, unclassified *Bergeyella* species, *Conchiformibius* sp. and *Porphyromonas cangingivalis*, whereas in our study, *Porphyromonas* A *cangingivalis*, *Porphyromonas gulae*, *Conchiformibius steedae*, *Porphyromonas* A *canoris* and *Porphyromonas gingivicanis* were the most abundant. Interestingly, *Pasteurella canis*, *Pasteurella multocida*, and *Bergeyella zoohelcum* were found in greater amounts in oral tumors in our study, but it is not possible to confirm that they are the same species in both studies because they were not classified in the previous study [16].

Genomics-based studies have shown that most of the cancers in humans also contain intratumoral microbiota [50,51,52,53], but there are no such studies in dogs. Intratumoral surface sampling in our study could lead to different results, but we intentionally swabbed the surface of the tumor along with all oral niches as we did in healthy dogs as our aim was to evaluate the overall microbiota (combining all oral niches) between the healthy and tumor groups.

In a study characterizing the oral microbiome in chronic ulcerative stomatitis in dogs (CCUS) using a 16S rRNA sequencing methodology, eight dogs with oral tumors were used as negative controls, along with dogs presented for dental procedures such as routine dental cleanings or treatment of severe periodontal disease [54]. The species that were more abundant in CCUS lesions were putative periodontal pathogens (*Porphyromonas cangingivalis* and *Porphyromonas gingivicanis*), as well as two canine species related to *Porphyromonas. gingivalis* and a *Tannerella forsythia*-like phylotype. The authors, Anderson et al., found some bacterial species more commonly associated with periodontitis, including putative periodontal pathogens (*Porphyromonas* spp., *Fusobacterium* spp., and *Prevotella* spp.). When comparing tumor surfaces with uninfected sites in CCUS animals, the authors found some marked differences, including the prevalence of three *Porphyromonas* species in tumor sites [54]. At the species level, in our study, *Porphyromonas cangingivalis* was the most abundant species among the phylum Bacteroidota in tumor patients, followed by *Porphyromonas gulae*, *Porphyromonas canoris*, and *Porphyromonas gingivicanis*, whereas among Fusobacteria, *Fusobacterium canifelinum* was the most abundant. However, there was no significant difference in the abundance of these species between healthy and oral tumor patients.

In another recent study, the correlation between the development of mammary tumors in dogs and the oral microbiota was investigated. For this purpose, oral swabs were taken by swabbing the lateral oral gingival mucosa of both healthy dogs and those that had developed mammary tumors. The authors, Zheng et al., concluded that the higher abundance of *Treponema* and *Bacteroides* could potentially serve as specific risk factors for the development and/or progression of mammary tumors [36]. However, in our study, we did not observe any differences in abundance of these bacterial taxa between healthy dogs and dogs with oral tumors. This discrepancy may stem from differences in the sampling method as the authors in the mentioned study only sampled the lateral gingival mucosa, whereas in our study all oral niches were included in a precise sampling sequence.

Recently, a study similar to ours was conducted in dogs with oral malignant melanomas, comparing their oral microbiota with that of a healthy control group to determine the risk factors for the development of these tumors [37]. The most abundant bacterium in both this and our study was *Porphyromonas cangingivalis*, and in both studies this species was found in significantly higher amounts in the groups with oral tumors. *Bergeyella zoohelcum* was significantly decreased in oral tumor group in the Carvalho et al. study [37], while it was increased in our study. Explaining the observed differences between the two studies is challenging as many factors can contribute to shifts in the oral microbiota. A significant factor is diet, which, similar to measures for oral home care, is difficult to control in a short-term clinical study. A recent report has shown that the relative abundances of *Fusobacteria* and over 20 bacterial genera were different in supragingival samples from dogs fed wet and dry diets [55]. In our study, most dogs were fed commercial food (dry, wet or combined), with a few receiving home-cooked meals. In the canine oral melanoma study [37], the distribution of dogs fed a certain diet among the included dogs was very similar. In general, bacterial taxa associated with oral health (such as *Pasteurella*, *Capnocytophaga*, *Corynebacterium*) were more abundant, while bacteria associated with poor oral health (including *Fretibacterium fastidiosum*, *Filifactor alocis*, *Treponema medium*, *Tannerella forsythia*, *Porphyromonas canoris*, *Porphyromonas gingivalis*) were less abundant in dogs fed dry food [55]. Such changes in the bacterial population within the oral microbiota may pose a risk for periodontal disease [55]. Unfortunately, the status of periodontal disease was not assessed in healthy dogs in our study, as the swabs were obtained from awake animals, while diagnosing the extent and severity of periodontal disease requires a detailed examination and radiography under general anesthesia. In the study published by Carvalho et al. [37], periodontal disease was assessed by the inspection of the oral cavity and periodontal spaces. A visual dental scale according to Bauer et al. [56] was used and periodontal disease was graded 0 to 4. In addition, samples of saliva were taken by rubbing the swab on the patients’ oral mucosa, further limiting the comparison between the two studies.

The different results observed between the oral melanoma study [37] and our study could also be attributed to antibiotic usage, known to have a significant impact on the fecal microbiota [43] and likely also oral microbiota, as suggested for other species (mice and rats) but not yet evaluated in dogs [44,45]. In the melanoma study [37], antibiotic use was either not controlled or the status of the animals was unknown. In contrast, all animals in our study had either ceased or were without antibiotic use at least two months prior to sampling. The same applies to the use of proton pump inhibitors, which have been shown to affect the fecal microbiota of both cats [46] and dogs [47] and also has an impact on the oral microbiome of humans [48]. Consequently, all dogs in our study did not take proton pump inhibitors for at least two months prior to sampling, although this was not part of the inclusion criteria in the oral melanoma study [39]. The same applies to the intake of glucocorticoids. All subjects in our study had not taken corticosteroids for a minimum of two months prior to sampling. A recent study in dogs investigated the effects of glucocorticoids on the mucosal microbiota in canine inflammatory bowel disease, concluding that the spatial distribution of mucosal bacteria was significantly different in dogs with inflammatory bowel disease (IBD) after prednisone therapy [49].

In our study, two bacterial genera previously described as being associated with oral health, *Pasteurella* and *Corynebacterium* [57], were found in higher quantities in the healthy group. *Porphyromonas canoris*, linked with poor oral health [57], was highly frequent in all samples, particularly in the group with oral tumors, contrasting the findings of similar studies conducted in humans. Namely, decreases in *Firmicutes* and increases in *Fusobacteria* were associated with OSCC in humans, but our study shows the opposite trend [24,25]. The increase in *Porphyromonas gingivalis* and *Fusobacterium nucleatum* in neoplastic tissue compared to normal tissue, correlated with subgingival plaque, suggests a potential close relationship between oral microorganisms, especially periodontal pathogens, and OSCC [58]. Infection with *Porphyromonas gingivalis* was associated with late clinical stage, low differentiation, and lymph node metastasis in OSCC patients, associated with deeper periodontal pockets, severe clinical attachment loss, and tooth loss [58]. In our study, we found no significant differences in the abundance of *Porphyromonas gingivalis* between the groups, whereas unspecified *Fusobacteria* were more abundant in healthy dogs. This further emphasizes that human and canine oral microbiota differ not only in health, but also in disease. In humans, *Fusobacterium nucleatum* is commonly found in the oral cavity and has become the focus of scientific interest over the past decade as more associations with extraoral disease emerge [59]. The role of *Fusobacterium nucleatum* as a carcinogenic member of the microbiota is still emerging and reveals the multiple ways in which a bacterium can contribute to the development, growth, spread, and response to treatment of cancer [59]. In a human study, researchers also established that a specific biofilm forms on the dorsum of the tongue, consisting mainly of *Fusobacterium nucleatum and Streptococcus* spp., believed to play an important role in intra-oral halitosis, and thus a potential target for treatment [60]. In dogs, *Fusobacterium* is a member of the normal oral microbiota [16,17,18,19]. In our study, among the *Fusobacterium* species, *Fusobacterium* sp. was the most abundant, followed by *Fusobacterium russii* and *Fusobacterium canifelinum.*

Another factor we should keep in mind is the stage and invasiveness of a tumor, which can impact the oral microbiota. A recent study [53] on oral tumors in humans, using 16S rRNA gene sequencing, found that the proportion of *Fusobacterium* and *Haemophilus* in the T3/T4 stages, in cases with lymphatic metastasis and in the group with extranodal expansion (where tumor cells penetrate the lymph node capsule into the perinodal tissue), were enriched, whereas the proportion of *Neisseria* was greater in T1/T2 stages, in non-lymphoid metastasis, and in the extranodal expansion group. In addition, the genus *Porphyromonas* was more abundant in T1/T2 stages but also in the lymphatic metastasis group, suggesting that tumor stage and invasiveness may influence the microbiota [53]. In our study, we were unable to account for this factor due to the absence of data on the exact tumor size and invasiveness (staging), making it impossible to completely exclude this effect.

Since all tumor patients (but none of the healthy control dogs) were sampled under general anesthesia, possible effects of anesthesia on the oral microbiota should be considered, although they are likely minimal or non-existent. Anesthesia may affect the gut microbiome [61], but there are currently no data for the oral microbiome. In the study conducted in rats, it was reported that the composition of the gut microbiome can be affected by exposure to anesthetics. However, changes were examined after a four-hour exposure to isoflurane, and the composition of the gut microbiome showed significant changes on day one and day seven after exposure. There was a decrease in bacterial α-diversity, an increase in the abundance of Proteobacteria and Actinobacteria, and a decrease in the abundance of Firmicutes and Clostridiales [62]. In a recent study, the authors examined the effect of sevoflurane inhalation anesthesia on species changes in the gut microbiome of mice and found that fecal samples collected on day 14 post-anesthesia had significantly increased abundances of *Bacteroides*, *Alloprevotella*, and *Akkermansia* and significantly decreased abundances of *Lactobacillus* compared with samples collected on day 1 post-anesthesia [63]. Another study using 16S rRNA sequencing yielded compelling results. It was performed with rats administered intravenously for three hours with the anesthetic propofol, which is used to induce anesthesia [64]. The abundance and diversity of intestinal flora in different time periods were compared and it was found that the continuous intravenous infusion of propofol had little effect on them. At the genus level, the diversity changed only in *Prevotella*, *Lactobacillus* and *Alloprevotella*, and to a lesser extent [64]. In the present study, samples were collected from all animals with oral tumors immediately after induction to anesthesia (before any other procedures), hence it can be assumed that the effect is minimal due to the short duration of anesthesia.

Limitations of this study include the small number of tumor patients due to the strict inclusion criteria; unfortunately, most dogs with an oral mass that were eligible for the study were treated with an antibiotic before presentation and were therefore excluded. An important limitation of our study was also the lack of inclusion of tumors of epithelial origin, making it difficult to compare our results with previous studies conducted in dogs [39], humans [22,23,24,30,31,32,33,58] or other species [35]. Additionally, the inability to control diet and home oral care in a clinical setting, and the unknown periodontal status, due to ethical concerns which prevented the use of anesthesia in healthy dogs, were further limitations. Furthermore, an age-, size-, and sex-matched group of healthy dogs was created based on the included dogs with oral tumors to minimize their possible effects. Because assigning breeds was not feasible, the size of the dogs was used as an approximate measure. Despite these limitations, this study contributes to establishing the core oral microbiota of healthy dogs, contributing to the library, which will hopefully soon be expanded. Additionally, this study shows that no major changes are seen in oral microbiota between the healthy and oral tumor patients, but that there are some differences at the species level that warrant further and more focused investigation.

## 5. Conclusions

In this study, we further characterized the oral microbiota of healthy dogs and dogs with oral tumors using next-generation sequencing. The results show that the oral microbiota is very diverse. In all dogs, 1032 bacterial species were detected, with 67 species representing the core microbiota, defined as the bacterial taxa present in all healthy dogs. There were no significant differences in species richness, bacterial communities, and specific taxa between different tumors. Our results suggest that oral tumors are not associated with fundamental alterations in oral microbiota. Further studies are needed to understand the role of the oral microbiota in the carcinogenesis of oral tumors in dogs.

## Figures and Tables

**Table 1:** Distribution of age (years), weight (kg), sex and breeds within the study population, categorized by healthy dogs and those with oral tumors.

	Healthy Group	Oral Tumor Group	*p*-Value
**Age (years)**			0.5026
Mean	7.5	8.3	
Min-Max	2–14.3	5.4–11.9	
**Weight (kg)**			0.3469
Mean	18.5	23.6	
Min-Max	3–42	6–50	
**Sex**			0.2015
Female (%)	13 (54.2)	1 (14.3)	
Male (%)	11 (45.8)	6 (85.7)	
**Breeds (n)**	Airedale Terrier, Beagle, Cavalier King Charles Spaniel, English Cocker Spaniel, French Bulldog (3), German Boxer (2), German Pointer, German Shepherd, German Spitz (3), Giant Schnauzer, Keeshond, Labrador retriever, Lagotto Romagnolo (2), Pomeranian, Russian Greyhound, Tibetan terrier (2), and Whippet	American Staffordshire Terrier, American Bulldog, Chihuahua, Cross-breed (2), Labrador Retriever, and Shih-Tzu	NA

NA: not applied.

**Table 2:** Relative abundance of identified phyla along with the range of abundance observed in healthy dogs.

Phylum	Median (%)	Range (%)
Bacteroidota	66.1	15.6–86.7
Proteobacteria	28.2	4.6–69.3
Actinobacteriota	2.1	0.3–12.5
Desulfobacterota	0.8	0.1–4.4
Firmicutes	0.8	0.2–11.1
Spirochaetota	0.5	<0.1–2.4
Firmicutes_A	0.4	0.1–1.1
Fusobacteriota	0.3	<0.1–0.6
Unclassified Phylum from Bacteria	0.2	0.1–5.6
Campylobacterota	0.2	<0.1–2.6
Patescibacteria	<0.1	0–0.2
Firmicutes_C	<0.1	0–0.5

Note: All values in the table have been rounded to one decimal place, with numbers greater than five rounded up.

**Table 3:** Relative abundance of most frequently identified species and range of abundance in healthy dogs.

Species	Median (%)	Range (%)
*Porphyromonas A cangingivalis*	15.9	4.9–31.3
*Porphyromonas gulae*	10.9	0.4–50.7
*Conchiformibius steedae*	6.2	0.7–40.4
*Porphyromonas A canoris*	4.1	0.5–23.9
*Porphyromonas gingivicanis*	4.0	0.1–13.2
*Neisseria Weaveri*	1.9	0.1–9.5
*Frederiksenia canicola*	1.6	0.2–17.2
*Capnocytophaga cynodegmi*	1.3	0.2–7.6
*Capnocytophaga canimorsus*	1.3	0.2–9.2
*Capnocytophaga canis*	1.1	0.4–5.5
*Bergeyella zoohelcum*	1.0	0.1–18.5
*Histophilus haemoglobinophilus*	0.9	0.3–18.6
*Pasteurella dagmatis*	0.9	0.0–17.3
*Neisseria zoodegmatis*	0.8	0.1–7.0
*Desulfomicrobium orale*	0.8	0.1–4.3
*Porphyromonas gingivalis*	0.8	0.1–3.1

Note: All values in the table have been rounded to one decimal place, with numbers greater than five rounded up.

**Table 4:** Core microbiota: median and range values based on the relative abundance of the bacterial species found in each individual sample from all healthy dogs.

Species	Median (%)	Range (%)
*Porphyromonas A cangingivalis*	15.87	4.88–31.34
*Porphyromonas gulae*	10.86	0.35–50.73
*Conchiformibius steedae*	6.18	0.65–40.44
*Porphyromonas A canoris*	4.05	0.51–23.85
*Porphyromonas gingivicanis*	3.98	0.12–13.22
*Neisseria weaveri*	1.89	0.08–9.48
*Frederiksenia canicola*	1.63	0.15–17.19
*Capnocytophaga cynodegmi*	1.30	0.16–7.64
*Capnocytophaga canimo rsus*	1.26	0.16–9.23
*Capnocytophaga canis*	1.08	0.39–5.48
*Bergeyella zoohelcum*	1.01	0.09–18.49
*Histophilus haemoglobinophilus*	0.91	0.32–18.57
*Pasteurella dagmatis*	0.91	0.02–17.30
*Neisseria zoodegmatis*	0.81	0.06–7.05
*Desulfomicrobium orale*	0.80	0.13–4.30
*Porphyromonas gingivalis*	0.80	0.13–3.13
*Porphyromonas* Other	0.71	0.10–2.82
*Pasteurella canis*	0.59	0.13–2.21
*Porphyromonas crevioricanis*	0.57	0.01–4.04
*Pasteurella multocida* A	0.55	0.01–3.16
Unidentified species from Order Bacteroidales	0.54	0.10–1.54
*Neisseria animaloris*	0.50	0.08–13.69
*Actinomyces* GCF 016598775.1	0.49	<0.01–2.40
*Capnocytophaga* Other	0.48	0.08–2.03
Unidentified species from Family *Porphyromonadaceae*	0.47	0.04–0.83
*Neisseria* Other	0.45	0.02–2.77
*Eikenella shayeganii*	0.37	0.07–3.20
*Tannerella forsythia*	0.31	0.04–0.93
Unidentified species from Family *Pasteurellaceae*	0.30	0.05–1.16
*Mycoplasmopsis A canis*	0.30	0.02–3.83
*Neisseria canis*	0.23	0.01–1.95
Other	0.20	0.1–5.57
*Treponema B denticola*	0.17	0.01–0.79
Unidentified species from Family *Neisseriaceae*	0.14	0.01–0.53
*Porphyromonas circumdentaria*	0.14	0.02–0.24
*Prevotella* Other	0.11	0.01–0.22
Unidentified species from Family *Bacteroidaceae*	0.11	0.01–0.26
*Treponema B* Other	0.11	0.01–0.39
*Moraxella* Other	0.09	0.03–0.29
*Corynebacterium mustelae*	0.09	0.01–4.69
*Fusobacterium* Other	0.07	<0.01–0.18
*Streptococcus minor*	0.06	0.01–2.00
*Gemella palaticanis*	0.06	0.01–0.67
*Pasteurella* Other	0.06	0.02–0.66
Unidentified species from Class Gammaproteobacteria	0.06	0.02–0.99
*Moraxella canis*	0.06	0.01–0.88
Unidentified species from Family *Moraxellaceae*	0.05	0.01–0.12
Unidentified species from Order Flavobacteriales	0.05	0.01–0.48
*Porphyromonas A* Other	0.05	<0.01–0.20
*Prevotella intermedia*	0.05	0.01–0.33
*Streptococcus* Other	0.04	<0.01–4.67
Unidentified species from Class Bacteroidia	0.04	0.01–0.10
*Neisseria wadsworthii*	0.04	<0.01–0.35
*Capnocytophaga stomatis*	0.04	0.01–3.18
*Campylobacter A* Other	0.04	<0.01–0.17
*Bacteroides* Other	0.03	<0.01–1.86
Unidentified species from Class Clostridia	0.03	0.01–0.06
Unidentified species from Family *Campylobacteraceae*	0.02	<0.01–0.10
Unidentified species from Order Enterobacterales	0.02	<0.01–0.07
*Histophilus somni*	0.02	<0.01–0.17
*Actinomyces* Other	0.01	<0.01–0.27
*Pasteurella multocida*	0.01	<0.01–0.48
*Treponema* Other	0.01	<0.01–0.12
*Acinetobacter* Other	0.01	<0.01–0.05
*Pauljensenia* Other	0.01	<0.01–0.04
Unidentified species from Order Burkholderiales	0.01	<0.01–0.04
Unidentified species from Class Actinomycetia	0.01	<0.01–0.10

Note: All values in the table have been rounded to two decimal places, with numbers greater than five rounded up.

## Data Availability

The raw data supporting the conclusions of this article will be made available by the authors, without undue reservation.

## Supplementary Materials

The following supporting information can be downloaded at: https://www.mdpi.com/article/10.3390/ani13233594/s1. Supplementary Table S1: Questions asked to the owners of the dogs included in the study, Supplementary Table S2: Baseline data of all patients included in the study (gender, age, weight), Supplementary Table S3: Median and relative abundance of bacterial taxa in healthy dogs, dogs with oral tumors and comparison between them at phylum, class, order, family, genus and species level, Supplementary Figure S1: Heat map representing the relative abundance of bacterial taxa at class level in healthy dogs and those with oral tumors, Supplementary Figure S2: Heat map representing the relative abundance of bacterial taxa at order level in healthy dogs and those with oral tumors, Supplementary Figure S3: Heat map representing the 25 most abundant bacterial taxa at order level in healthy dogs and those with oral tumors, Supplementary Figure S4: Heat map representing the relative abundance of bacterial taxa at family level in healthy dogs and those with oral tumors.
