# Supplementary material for: A Comparison of the Oral Microbiota in Healthy Dogs and Dogs with Oral Tumors
Source: Animals (Basel). 2023 Nov 21;13(23):3594. doi: 10.3390/ani13233594 (PMC10705671; doi:10.3390/ani13233594)
Supplement: Supplementary file 1 [file animals-13-03594-s001.zip › Supplementary Figures.pdf]

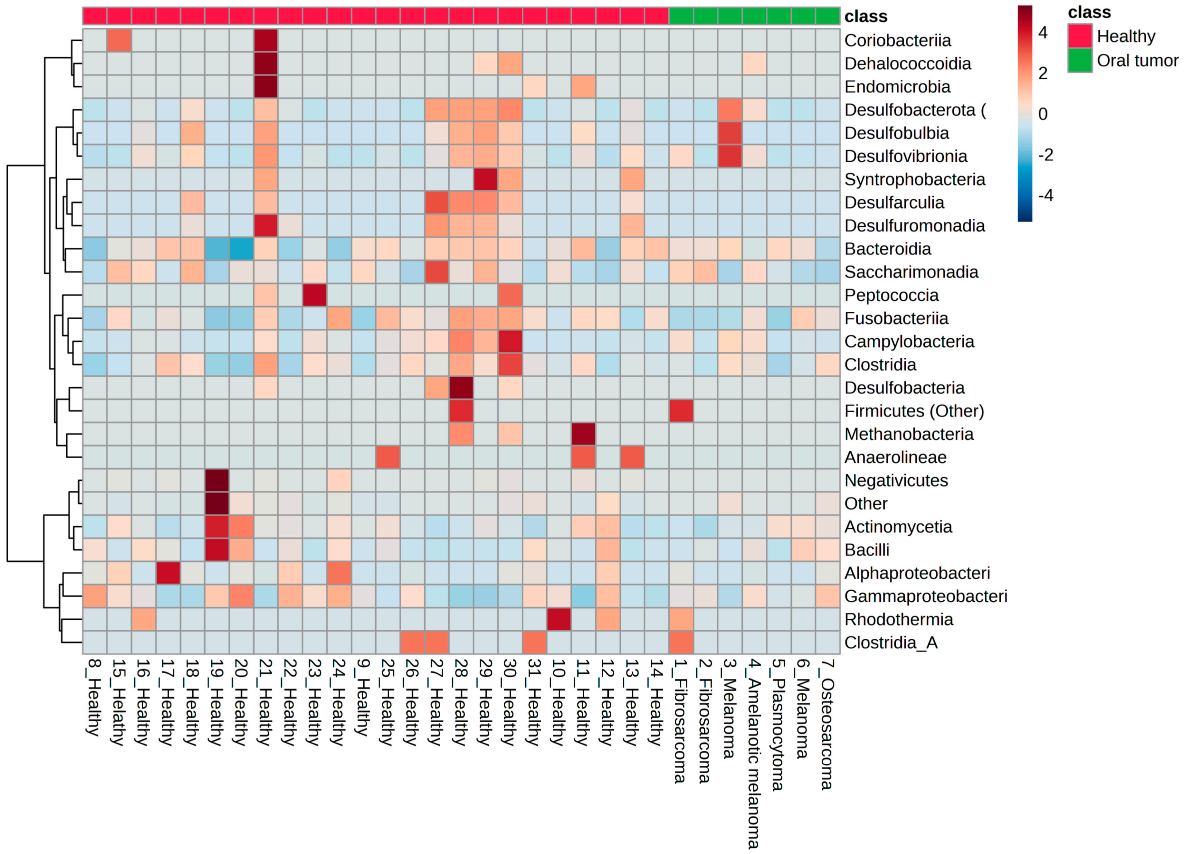

Figure S1. Heat map representing the relative abundance of bacterial taxa at class level in healthy dogs and those with oral tumors.

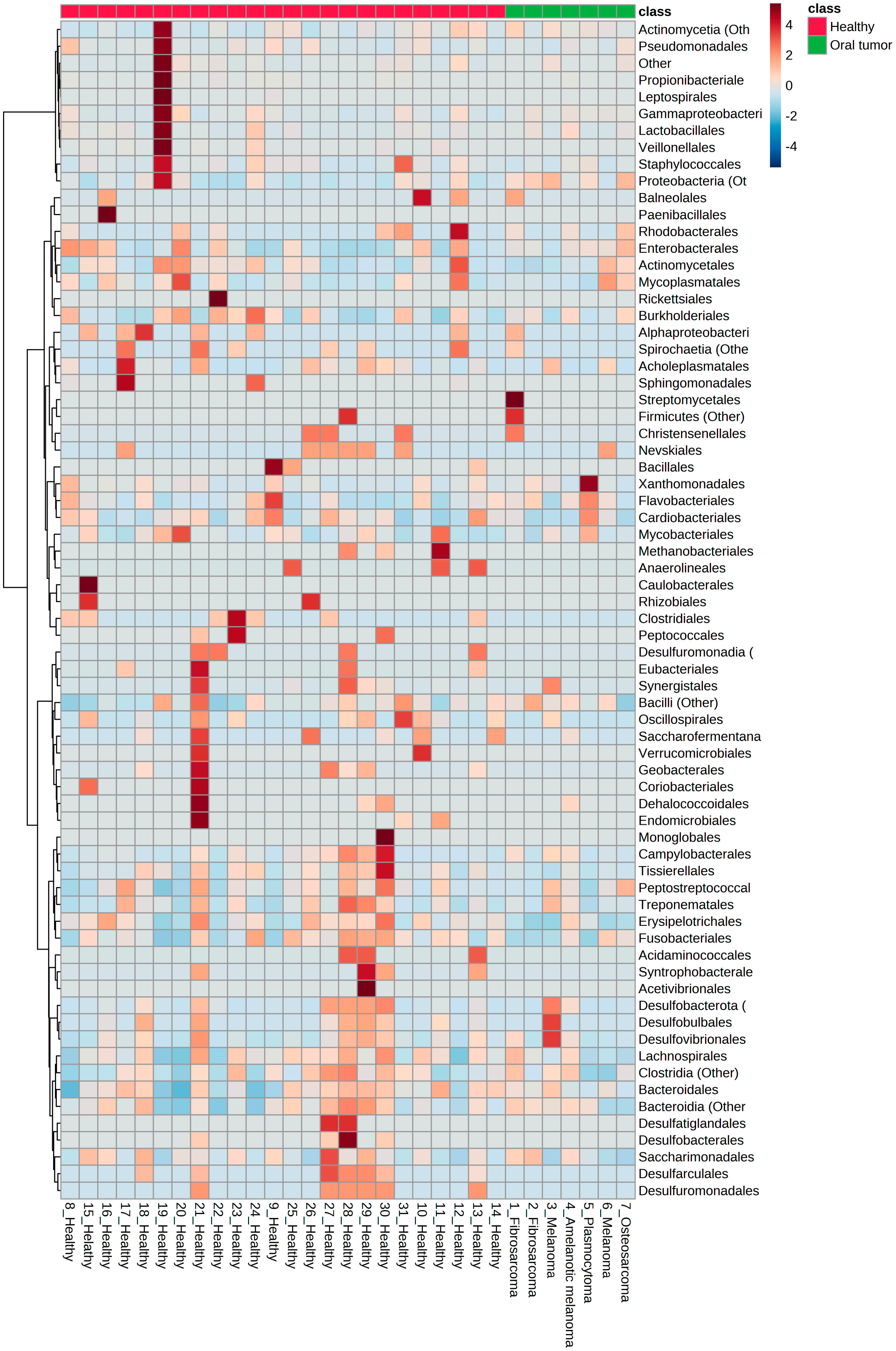

Figure S2. Heat map representing the relative abundance of bacterial taxa at order level in healthy dogs and those with oral tumors.

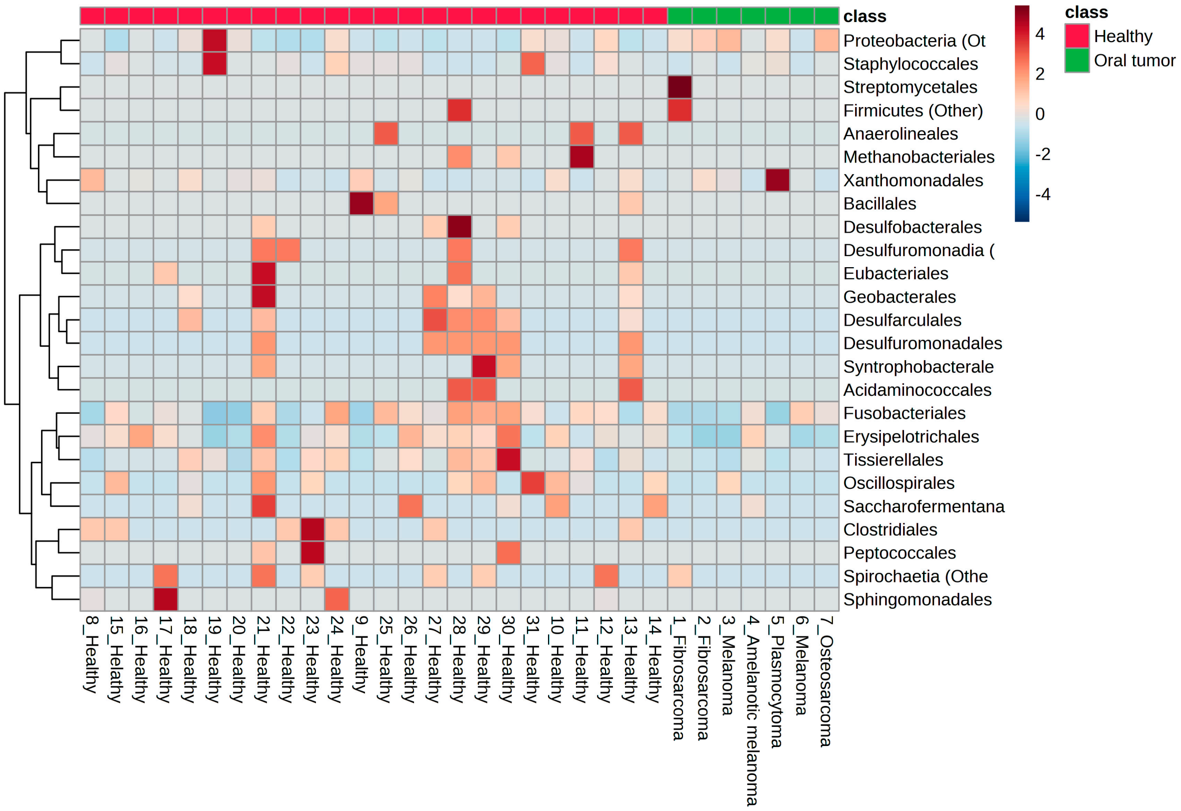

Figure S3. Heat map representing the 25 most abundant bacterial taxa at order level in healthy dogs and those with oral tumors.

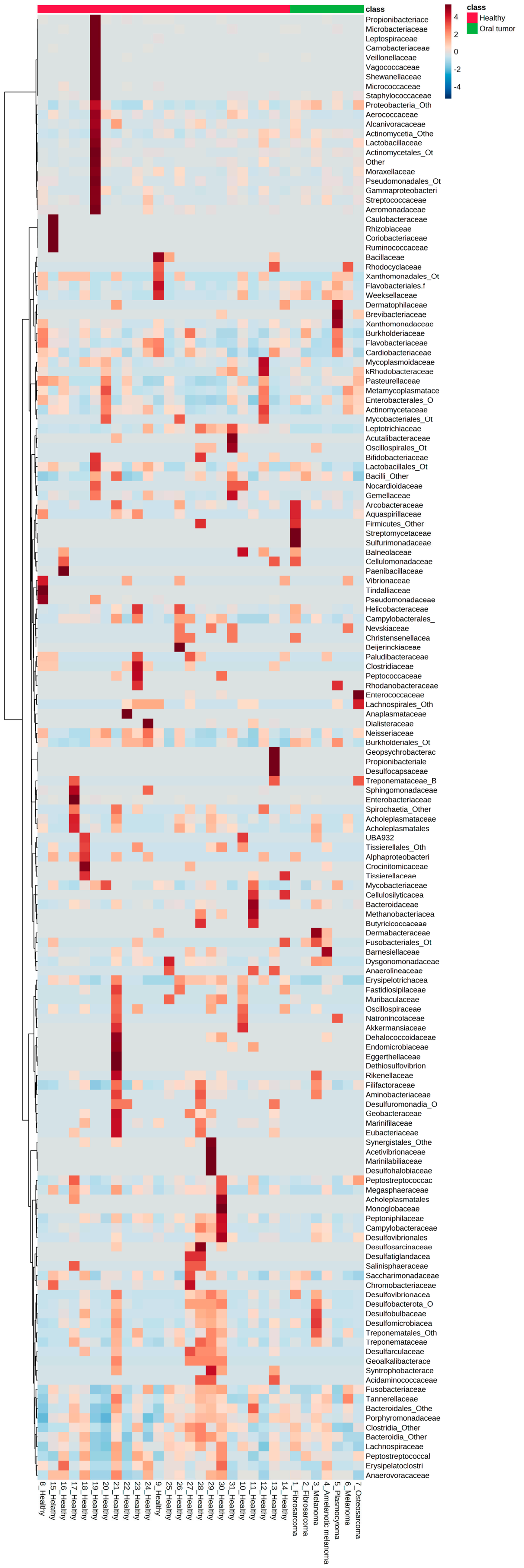

Figure S4: Heat map representing the relative abundance of bacterial taxa at family level in healthy dogs and those with oral tumors.
