# Supplementary material for: A Comparison of the Oral Microbiota in Healthy Dogs and Dogs with Oral Tumors
Source: Animals (Basel). 2023 Nov 21;13(23):3594. doi: 10.3390/ani13233594 (PMC10705671; doi:10.3390/ani13233594)
Supplement: Supplementary file 1 [file animals-13-03594-s001.zip › Supplementary Table S1.pdf]

Supplementary Table S1. Questions asked to the owners of the dogs included in the study.

QUESTION	HEALTHY				ORAL TUMOUR			
**Type of food?**	Dry	Wet	Dry and wet	Home-cooked	Dry	Wet	Dry and wet	Home-cooked
	11	0	10	2	1	0	5	1
**Treats?**	Yes		No		Yes		No	
	19		5		4		3	
**Vitamins or other supplements?**	Yes		No		Yes		No	
	6		18		2		5	
**Antipasitics?**	Yes		No		Yes		No	
	24		0		0		7	
**Antibiotics?**	Yes		No		Yes		No	
	0		24		0		7	
**Immunosuppressive drugs?**	Yes		No		Yes		No	
	0		24		0		7	
**Gastroprotectants?**	Yes		No		Yes		No	
	0		24		0		7	
**Product for oral care containing clorhexidine?**	Yes		No		Yes		No	
	2		22		1		6	
**Previous dental procedure?**	Yes		No		Yes		No	
	3		21		2		5	
**Do you brush teeth?**	Yes		No		Yes		No	
	5		19		2		5	
**Other oral care/plaque removal products?**	Yes		No		Yes		No	
	10		14		2		5	
