# Supplementary material for: A Comparison of the Oral Microbiota in Healthy Dogs and Dogs with Oral Tumors
Source: Animals (Basel). 2023 Nov 21;13(23):3594. doi: 10.3390/ani13233594 (PMC10705671; doi:10.3390/ani13233594)
Supplement: Supplementary file 1 [file animals-13-03594-s001.zip › Supplementary Table S2.pdf]

Table S2. Baseline data of all patients included in the study (gender, age, weight).

PATIENTS	BREED	SEX	AGE (months)	WEIGHT (kg)
**Healthy**	German Pointer	M	86	30
	Giant Schnauzer	F	24	31
	Airedale Terrier	FS	120	26
	Cavalier King Charles spaniel	FS	125	7
	English Cocker Spaniel	FS	140	14
	Tibetan terrier	M	77	12,5
	Whippet	MN	75	17,7
	German Spitz	M	64	6,4
	Lagotto Romagnolo	FS	60	12,5
	Beagle	FS	152	27
	German Shepherd	FS	72	28
	Lagotto Romagnolo	M	109	20
	Russian Greyhound	M	51	42
	Tibetan terrier	M	172	13
	Labrador retriever	F	95	28
	French bulldog	M	94	14
	French bulldog	FS	93	11,8
	French bulldog	FS	88	13
	Keeshond	F	67	16
	German Spitz	M	64	5
	German Spitz	F	115	5
	Pomeranian	F	63	3
	German boxer	M	100	30
	German boxer	M	53	32
**Fibrosarcoma**	Labrador Retriever	FS	130	26
**Fibrosarcoma**	Cross-breed	M	81	40
**Melanoma**	Cross-breed	FS	65	9
**Amelanotic melanoma**	Chihuahua	FS	143	7
**Plasmacytoma**	America Staffordshire Terrier	F	90	27
**Melanoma**	Shih-Tzu	F	103	6
**Osteosarcoma**	American Bulldog	FS	86	50

M= male, MN= male neutered, F= female, FS= female spayed
